# Healthcare resource utilization and costs in patients with a newly confirmed diagnosis of lupus nephritis in the United States over a 5-year follow-up period

**DOI:** 10.1186/s12913-024-11060-6

**Published:** 2024-05-31

**Authors:** Christopher F. Bell, Benjamin Wu, Shirley P. Huang, Bernard Rubin, Carlyne M. Averell, Benjamin Chastek, Erin M. Hulbert

**Affiliations:** 1 GSK, US Value, Evidence and Outcomes, 410 Blackwell Street, Durham, NC 27701 USA; 2 GSK, US Medical Affairs and Immuno-Inflammation, Durham, NC USA; 3 Optum, Life Sciences, Eden Prairie, MN USA

**Keywords:** Nephritis, Renal lupus, Systemic lupus erythematosus, Lupus nephritis, Healthcare resource utilization, Healthcare cost

## Abstract

**Background:**

We aimed to describe healthcare resource utilization (HCRU) and healthcare costs in patients with newly confirmed lupus nephritis (LN) in the United States over a 5-year follow-up period.

**Methods:**

This retrospective, longitudinal cohort study (GSK Study 214102) utilized administrative claims data to identify individuals with a newly confirmed diagnosis of LN between August 01, 2011, and July 31, 2018, based on LN-specific International Classification of Diseases diagnosis codes. Index was the date of first LN-related diagnosis code claim. HCRU, healthcare costs, and incidence of systemic lupus erythematosus (SLE) flares were reported annually among eligible patients with at least 5 years continuous enrollment post-index.

**Results:**

Of 2,159 patients with a newly confirmed diagnosis of LN meeting inclusion and exclusion criteria, 335 had at least 5 years continuous enrollment post-index. HCRU was greatest in the first year post-LN diagnosis across all categories (inpatient admission, emergency room [ER] visits, ambulatory visits, and pharmacy use), and trended lower, though remained substantial, in the 5-year follow-up period. Among patients with LN and HCRU, the mean (standard deviation [SD]) number of ER visits and inpatient admissions were 3.7 (4.6) and 1.8 (1.5), respectively, in Year 1, which generally remained stable in Years 2–5; the mean (SD) number of ambulatory visits and pharmacy fills were 35.8 (25.1) and 62.9 (43.8), respectively, in Year 1, and remained similar for Years 2–5. Most patients (≥ 91.6%) had ≥ 1 SLE flare in each of the 5 years of follow-up. The proportion of patients who experienced a severe SLE flare was higher in Year 1 (31.6%) than subsequent years (14.3–18.5%). Total costs (medical and pharmacy; mean [SD]) were higher in Year 1 ($44,205 [71,532]) than subsequent years ($29,444 [52,310]–$32,222 [58,216]), driven mainly by inpatient admissions (Year 1: $21,181 [58,886]; subsequent years: $7,406 [23,331]–$9,389 [29,283]).

**Conclusions:**

Patients with a newly confirmed diagnosis of LN have substantial HCRU and healthcare costs, particularly in the year post-diagnosis, largely driven by inpatient costs. This highlights the need for improved disease management to prevent renal damage, improve patient outcomes, and reduce costs among patients with renal involvement.

**Supplementary Information:**

The online version contains supplementary material available at 10.1186/s12913-024-11060-6.

## Background

Systemic lupus erythematosus (SLE) is a chronic autoimmune disease characterized by autoantibody production and abnormal immunological response [1, 2]. SLE can affect multiple organs and systems, including musculoskeletal, dermatologic, neuropsychiatric, hematologic, renal, and cardiovascular [3, 4]. Common symptoms experienced by patients include fatigue/weakness and joint pain/swelling [5].

The goals of SLE treatment, in addition to the control of disease activity and prevention of new flares, include improving patient health-related quality of life (HRQoL), preventing organ damage, and improving long-term patient survival [3]. Treatment strategies for the management of SLE include the use of antimalarials, corticosteroids, immunosuppressants, and biologics (e.g., belimumab) [3]. Corticosteroids remain the mainstay of treatment for the short-term control of flares; however, cumulative use is associated with dose-dependent and irreversible organ damage [6, 7] which further contributes to an increased risk of death [8].

Lupus nephritis (LN), a form of glomerulonephritis, is one of the most severe complications of SLE [9]. Approximately 40% of patients with SLE develop LN, which can lead to development of chronic kidney disease, acute kidney injury, and eventually progression to end-stage kidney disease in 5–20% of patients with LN within 10 years of SLE diagnosis [9, 10]. A single LN flare can result in irreversible nephron loss, which may shorten the kidney lifespan by decades [9].

Considering the chronic disease course, increased morbidity and mortality, and poor HRQoL associated with SLE, inclusive of LN [11, 12], studies evaluating healthcare resource utilization (HCRU) and healthcare costs are essential to understand the clinical and economic impact of the disease, as well as serving as a basis for cost-effectiveness analyses.

Patients with SLE have higher HCRU than patients without SLE [13–16]. A study reporting HCRU from electronic health records in 17,257 patients with SLE estimated that 94.4% of patients had ≥ 1 outpatient visit, 25.7% had ≥ 1 emergency room (ER) visit, and 13.7% had ≥ 1 inpatient admission on average per year [17].

SLE is associated with substantial annual direct costs, with pharmaceutical, inpatient, and outpatient services making a large contribution to total costs [18]. A real-world cohort study conducted in the United States estimated that the mean unadjusted all-cause healthcare cost of SLE was $33,897 in the year post-diagnosis [19]. Similarly, another retrospective claims analysis estimated that the mean total annual cost of SLE for patients who had ≥ 1 healthcare encounter was $32,374 [17]. Costs have been shown to increase with disease severity; adjusted mean total healthcare costs (excluding pharmacy costs) were estimated to be $39,021 for patients with moderate/severe SLE and $23,519 for mild SLE among commercially insured patients [20]. Costs among patients with SLE have also been shown to generally increase over time [21]. SLE flare frequency and severity contributes considerably to healthcare costs, with a severe flare costing approximately $27,468 in the 90 days after the flare [22].

It is well established that HCRU and healthcare costs are substantially higher in patients with LN compared with patients with SLE without LN, or controls [21, 23–28], with medical costs specific to LN reportedly accounting for approximately 41% of total medical costs in patients with SLE [29]. One study demonstrated that annual costs for patients with LN were 155% higher than for patients with SLE alone [23]. Another study reported a near doubling of total all-cause healthcare costs in patients with LN compared with patients with SLE without LN, with inpatient costs approximately three times higher [28].

A previous longitudinal analysis of a Medicaid population assessed healthcare costs and HCRU in patients with SLE and LN compared with SLE alone over a 5-year period (spanning 1999–2005) [21]; analyses of commercially insured and Medicare populations would provide further insights into the burden of LN in the United States. Additionally, despite the high burden of LN in patients with SLE, there is limited recent longitudinal data on HCRU and healthcare costs among newly diagnosed patients with LN over time. Accordingly, the objective of this longitudinal study was to describe HCRU and healthcare costs in commercial and Medicare Advantage insurance plan members with a newly confirmed diagnosis of LN in the United States over a 5-year follow-up period.

## Methods

### Study design

This retrospective, longitudinal cohort study (GSK Study 214102) was conducted using the Optum Research Database of commercial and Medicare Advantage insurance plan members in the United States.

**Fig. 1:**
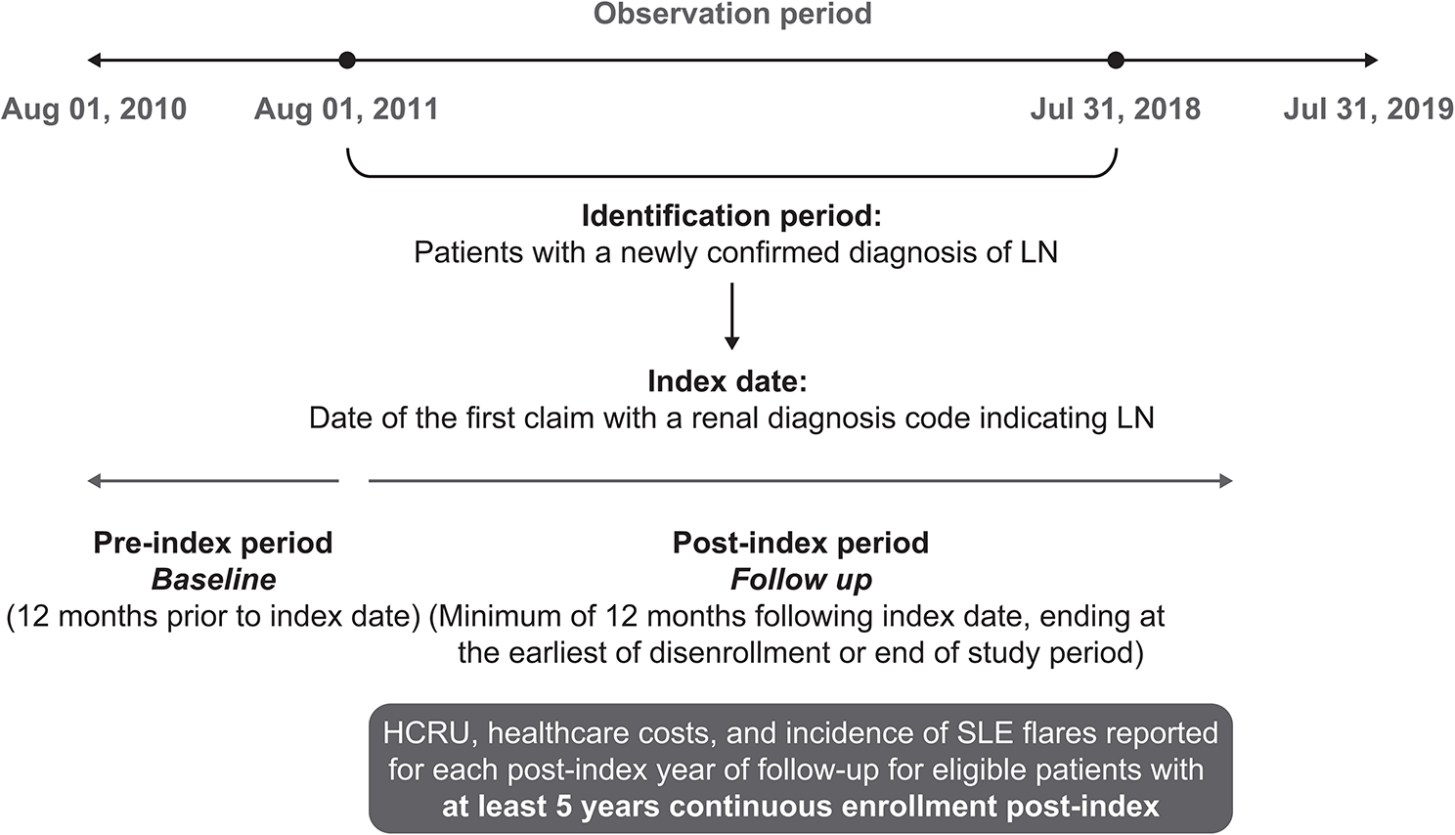
Study period and data collection schematic HCRU, healthcare cost utilization; LN, lupus nephritis; SLE, systemic lupus erythematosus

The observation period spanned from August 01, 2010, to July 31, 2019 (Fig. 1). Commercial and Medicare Advantage insurance plan members with a newly confirmed diagnosis of LN between August 01, 2011, and July 31, 2018 (identification period), were included. The cohort was identified using a modified version of the algorithm reported by Chibnik and colleagues [30] to include renal-related International Classification of Diseases (ICD)-9 and ICD-10 codes indicative of LN. The index date was defined as the date of the first claim with a renal diagnosis code indicating LN during the identification period. Pre- and post-index periods were defined as the 12 months prior to the index date (baseline) and a minimum of 12 months following the index date, respectively. The follow-up end date was defined as the date of disenrollment or end of the study period, whichever occurred earliest. The current longitudinal analysis focuses on the subset of eligible patients with at least 5 years of continuous enrollment post-index, presenting a 5-year longitudinal view of patients with a newly confirmed diagnosis of LN.

### Study population

For inclusion in the overall population, patients were required to be aged ≥ 18 years during the year of index, have had ≥ 2 medical claims with renal diagnosis codes (ICD-10 codes that start with N00-N08, N17, N18, N19, R80, M32.14, or M32.15, *or* ICD-9 codes 580–586, 791) during the identification period (≥ 30 days apart and the second claim within 6 months of the first), have had ≥ 1 inpatient SLE diagnosis code or ≥ 2 SLE diagnosis codes (ICD-10 codes that start with M32, M32.0, M32.1, M32.10, M32.11, M32.12, M32.13, M32.14, M32.15, M32.19, M32.8, or M32.9, *or* ICD-9 code 710.0) in any setting during the pre-index period, and have had continuous enrollment with medical and pharmacy benefits of ≥ 12 months pre- and post-index.

Patients were excluded from the study if they had an LN diagnosis during the 12-month pre-index period; invalid demographic information; or ICD-9 or ICD-10 diagnosis codes indicating drug-induced SLE, pregnancy, human immunodeficiency virus, or acquired immunodeficiency syndrome during the identification period.

### Outcomes

HCRU, treatment patterns, SLE flares, and healthcare costs were reported for each post-index year of the 5 years of follow-up. Year 1 outcomes included the index date. Treatment patterns were reported for each post-index year of the 5 years of follow-up in 6-month intervals. Pharmacy claims were used for analysis of medications and to calculate prednisone-equivalent corticosteroid dose (dose/time period).

HCRU included inpatient admissions, ER visits, ambulatory visits (physician office and hospital outpatient), and pharmacy use (≥ 1 dispensing).

The number and severity of SLE flares were reported for each post-index year of follow-up based on the algorithm published by Garris et al. [31].

Healthcare costs were calculated as the sum of health plan and patient paid amounts and adjusted using the Consumer Price Index to reflect inflation to the year 2019. Specifically, total costs were calculated and reported separately for medical costs and pharmacy costs. Medical costs included costs of inpatient admissions, ER visits, ambulatory visits (physician office and hospital outpatient), and other services (including costs for services rendered at independent laboratories, at urgent care clinics, and by home health providers).

### Statistical analysis

Study variables, including pre-index and post-index outcome measures, were descriptive in nature.

Baseline demographic and clinical characteristics were analyzed in the cohort of patients with a newly confirmed diagnosis of LN at baseline and at least 5 years of follow-up data, filtered from all patients meeting the overall study inclusion and exclusion criteria.

Longitudinal outcomes were descriptively analyzed by each year of follow-up post-index in the subset of incident LN patients with at least 5 years of follow-up. Mean and standard deviation (SD) were provided for continuous variables.

### Ethical approval

All database records were de-identified and fully compliant with US patient confidentiality requirements, including the Health Insurance Portability and Accountability Act of 1996. The study used only de-identified patient records and did not involve the collection, use, or transmittal of individually identifiable data; thus, Institutional Review Board approval was not pursued, and informed consent was not required.

## Results

### Baseline patient demographics and clinical characteristics

The mean (SD) patient follow-up time was 1,100 (637) days. In this analysis, the study outcomes of HCRU and costs were evaluated for the subset of 335 patients (15.5%) with a newly confirmed diagnosis of LN who had at least 5 years of continuous enrollment post-index.

Among the 335 patients with a newly confirmed diagnosis of LN during the 12 months pre-index and at least 5 years of continuous enrollment post-index the mean (SD) age was 57.2 (14.1) years, and the majority were female (*n* = 294/335; 87.8%) and from the south region of the United States (*n* = 157/335; 46.9%). Approximately half of the patient population (*n* = 172/335; 51.3%) had Medicare Advantage insurance and the other half (*n* = 163/335; 48.7%) were commercially insured (Table 1). Overall, 24.2% (*n* = 81/335) of patients received their index diagnosis of LN from a nephrologist, 10.7% (*n* = 36/335) from a rheumatologist, and 34.3% (*n* = 115/335) from a primary care physician/family practitioner/internal medicine physician, with the remaining 30.7% (*n* = 103/335) receiving their diagnoses from an ‘other’ provider. Patients had a mean (SD) baseline Quan-Charlson comorbidity score of 2.1 (1.5) [32].

**Table 1:** Baseline demographics and disease characteristics in patients with ≥ 5 years of continuous enrollment post-index (*n* = 335)

	Patients
Age, mean (SD)	57.2 (14.1)
Age group, n (%)	
18–44	61 (18.2)
45–64	166 (49.6)
≥ 65	108 (32.2)
Female, n (%)	294 (87.8)
Region, n (%)	
Northeast	43 (12.8)
Midwest	80 (23.9)
South	157 (46.9)
West	55 (16.4)
Insurance type, n (%)	
Commercial	163 (48.7)
Medicare Advantage	172 (51.3)
Index year, n (%)	
2011	51 (15.2)
2012	118 (35.2)
2013	110 (32.8)
2014	56 (16.7)
Index LN diagnosis provider specialty, n (%)	
Rheumatologist	36 (10.7)
Nephrologist	81 (24.2)
Primary care physician/family practitioner/internal medicine physician	115 (34.3)
Other	103 (30.7)
Baseline Charlson comorbidity score, mean (SD)	2.1 (1.5)
Comorbidities,^a^ n (%)	
Hypertension	241 (71.9)
Nontraumatic joint disorders	214 (63.9)
Other connective tissue disease	199 (59.4)
Heart disease	194 (57.9)
Other lower respiratory disease	171 (51.0)
CKD staging, n (%)	
None	145 (43.3)
Stage I	37 (11.0)
Stage II	60 (17.9)
Stage III	83 (24.8)
Stage IV or V	10 (3.0)
Treatment, n (%)	
Corticosteroids	241 (71.9)
Oral	198 (59.1)
IV	114 (34.0)
Antimalarials	174 (51.9)
ACE inhibitors/ARBs	137 (40.9)
NSAIDs	121 (36.1)
Immunosuppressants	100 (29.9)
Azathioprine	36 (10.7)
Methotrexate	36 (10.7)
Mycophenolate^b^	37 (11.0)
Biologics	11 (3.3)
Belimumab	9 (2.7)

^a^Five most commonly reported comorbidities

^b^Mycophenolate: mycophenolate mofetil or mycophenolate sodium

ACE, angiotensin-converting enzyme; ARB, angiotensin receptor blocker; CKD, chronic kidney disease; IV, intravenous; LN, lupus nephritis; NSAID, nonsteroidal anti-inflammatory drug; SD, standard deviation

### HCRU

**Fig. 2:**
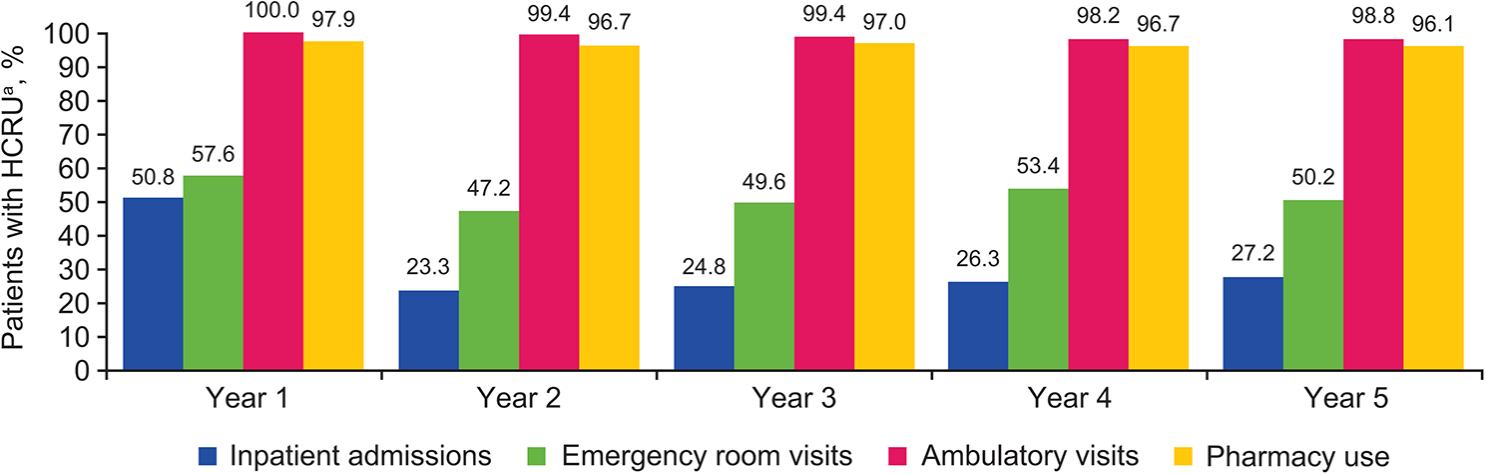
Longitudinal all-cause HCRU among patients with newly confirmed LN and ≥ 5 years of follow-up (*n* = 335) ^a^Patients newly diagnosed with LN with ≥ 5 years of follow-up and HCRU, %. HCRU, healthcare resource utilization; LN, lupus nephritis

Among the 335 patients with a newly confirmed diagnosis of LN with at least 5 years follow-up, HCRU was highest in the first year post-LN diagnosis across all categories (Fig. 2).

Approximately half of patients (*n* = 170/335; 50.8%) had ≥ 1 inpatient admission, and more than half of patients (*n* = 193/335; 57.6%) had ≥ 1 ER visit in Year 1; these proportions were lower in Years 2–5 (inpatient admissions: 23.3–27.2%; ER visits: 47.2–53.4%; Fig. 2). Among these patients, the mean (SD) number of inpatient admissions and ER visits was 1.8 (1.5) and 3.7 (4.6), respectively, in Year 1; the mean number of visits remained relatively stable in subsequent years of follow-up for both inpatient admissions and ER visits (Table 2).

**Table 2:** All-cause HCRU counts among patients with utilization and ≥ 5 years of follow-up (*n* = 335)

In patients with ≥ 1 event	Year 1	Year 2	Year 3	Year 4	Year 5
Inpatient admissions					
n	170	78	83	88	91
Number of visits					
Mean (SD)	1.8 (1.5)	1.7 (1.3)	1.9 (1.7)	1.8 (1.6)	1.9 (1.8)
Median (IQR)	1.0 (1.0–2.0)	1.0 (1.0–2.0)	1.0 (1.0–2.0)	1.0 (1.0–2.0)	1.0 (1.0–2.0)
Length of stay, days					
Mean (SD)	17.6 (23.2)	17.2 (22.3)	21.3 (26.8)	16.5 (20.5)	24.0 (29.1)
Median (IQR)	9.0 (5.0–20.0)	8.0 (4.0–19.0)	9.0 (5.0–30.0)	7.0 (4.0–19.5)	11.0 (4.0–32.0)
ER visits					
n	193	158	166	179	168
Number of visits					
Mean (SD)	3.7 (4.6)	3.7 (5.3)	3.9 (5.3)	3.6 (5.0)	4.1 (5.6)
Median (IQR)	2.0 (1.0–4.0)	2.0 (1.0–3.0)	2.0 (1.0–4.0)	2.0 (1.0–4.0)	2.0 (1.0–4.0)
Ambulatory visits^a^					
n	335	333	333	329	331
Number of visits					
Mean (SD)	35.8 (25.1)	31.4 (24.7)	31.3 (26.5)	33.0 (29.7)	32.3 (27.9)
Median (IQR)	30.0 (19.0–47.0)	24.0 (14.0–41.0)	24.0 (14.0–39.0)	23.0 (13.0–45.0)	25.0 (14.0–43.0)
Office visits					
n	333	331	328	326	328
Number of visits					
Mean (SD)	23.0 (17.5)	19.9 (16.0)	19.2 (15.1)	18.2 (14.3)	18.3 (14.1)
Median (IQR)	20.0 (12.0–28.0)	16.0 (9.0–26.0)	15.0 (9.0–24.0)	15.0 (9.0–24.0)	15.0 (9.0–23.5)
Outpatient visits					
n	297	286	280	279	290
Number of visits					
Mean (SD)	14.8 (14.3)	13.7 (16.1)	15.0 (20.0)	17.8 (24.7)	16.2 (22.6)
Median (IQR)	10.0 (5.0–19.0)	8.0 (4.0–18.0)	8.0 (4.0–18.0)	8.0 (4.0–21.0)	9.0 (4.0–19.0)
Pharmacy fills					
n	328	324	325	324	322
Number of fills					
Mean (SD)	62.9 (43.8)	60.7 (44.0)	59.7 (43.6)	59.9 (46.5)	58.0 (43.8)
Median (IQR)	54.0 (32.0–80.0)	48.0 (29.0–81.0)	47.0 (29.0–80.0)	47.0 (29.0–79.0)	45.0 (28.0–74.0)

^a^Includes physician office and hospital outpatient visits

ER, emergency room; HCRU, healthcare resource utilization; IQR, interquartile range; SD, standard deviation

The proportion of patients with an ambulatory visit or pharmacy use remained high (> 96.1%) across each of the 5 years of follow-up (Fig. 2). The mean (SD) number of ambulatory visits among patients with utilization in this category was 35.8 (25.1) in Year 1, and remained similar for Years 2–5 (Year 2, mean [SD]: 31.4 [24.7]; Year 3: 31.3 [26.5]; Year 4: 33.0 [29.7]; Year 5: 32.3 [27.9]; Table 2). The mean (SD) number of pharmacy fills among patients with utilization in this category across the 5 years of follow-up ranged from 58.0 (43.8) to 62.9 (43.8). The most frequently used SLE and LN treatments were corticosteroids (47.2–60.0%), antimalarials (40.6–51.0%), and angiotensin‐converting enzyme (ACE) inhibitors/angiotensin receptor blockers (ARBs) (40.6–44.5%; Table 3) with the proportion of patients with any treatment use being highest during the first 6-month interval with the exception of ACE inhibitors/ARBs (Table 3).

**Table 3:** Treatment counts among patients with ≥ 5 years of follow-up (*n* = 335)

Treatment, *n* (%)	Months 1–6	Months 7–12	Months 13–18	Months 19–24	Months 25–30	Months 31–36	Months 37–42	Months 43–48	Months 49–54	Months 55–60
NSAIDs	76 (22.7)	69 (20.6)	71 (21.2)	58 (17.3)	55 (16.4)	54 (16.1)	59 (17.6)	63 (18.8)	50 (14.9)	54 (16.1)
Antimalarials	171 (51.0)	160 (47.8)	150 (44.8)	157 (46.9)	158 (47.2)	146 (43.6)	151 (45.1)	147 (43.9)	144 (43.0)	136 (40.6)
Immunosuppressants	109 (32.5)	105 (31.3)	103 (30.7)	94 (28.1)	91 (27.2)	85 (25.4)	82 (24.5)	78 (23.3)	76 (22.7)	76 (22.7)
Methotrexate	30 (9.0)	28 (8.4)	26 (7.8)	22 (6.6)	20 (6.0)	16 (4.8)	15 (4.5)	15 (4.5)	15 (4.5)	15 (4.5)
Mycophenolate^a^	56 (16.7)	50 (14.9)	54 (16.1)	54 (16.1)	51 (15.2)	47 (14.0)	42 (12.5)	44 (13.1)	44 (13.1)	44 (13.1)
Cyclophosphamide	11 (3.3)	7 (2.1)	1 (0.3)	1 (0.3)	1 (0.3)	1 (0.3)	1 (0.3)	0 (0.0)	0 (0.0)	0 (0.0)
Azathioprine	22 (6.6)	26 (7.8)	27 (8.1)	23 (6.9)	23 (6.9)	26 (7.8)	25 (7.5)	20 (6.0)	19 (5.7)	20 (6.0)
Biologics	10 (3.0)	10 (3.0)	10 (3.0)	11 (3.3)	9 (2.7)	9 (2.7)	7 (2.1)	9 (2.7)	7 (2.1)	8 (2.4)
Belimumab	9 (2.7)	9 (2.7)	8 (2.4)	8 (2.4)	8 (2.4)	7 (2.1)	7 (2.1)	6 (1.8)	5 (1.5)	5 (1.5)
Rituximab	1 (0.3)	1 (0.3)	2 (0.6)	3 (0.9)	1 (0.3)	2 (0.6)	0 (0.0)	3 (0.9)	2 (0.6)	3 (0.9)
ACE inhibitors/ARBs	145 (43.3)	142 (42.4)	136 (40.6)	149 (44.5)	141 (42.1)	145 (43.3)	144 (43.0)	137 (40.9)	141 (42.1)	145 (43.3)
Corticosteroids	201 (60.0)	181 (54.0)	189 (56.4)	189 (56.4)	165 (49.3)	175 (52.2)	171 (51.0)	172 (51.3)	158 (47.2)	162 (48.4)
IV	78 (23.3)	72 (21.5)	66 (19.7)	86 (25.7)	57 (17.0)	78 (23.3)	79 (23.6)	69 (20.6)	62 (18.5)	68 (20.3)
Oral	174 (51.9)	145 (43.3)	155 (46.3)	155 (46.3)	138 (41.2)	131 (39.1)	131 (39.1)	135 (40.3)	126 (37.6)	132 (39.4)
≥ 5 mg/day^b^	163 (93.7)	136 (93.8)	141 (91.0)	142 (91.6)	126 (91.3)	121 (92.4)	119 (90.8)	121 (89.6)	113 (89.7)	119 (90.2)
≥ 7.5 mg/day^b^	141 (81.0)	108 (74.5)	112 (72.3)	111 (71.6)	97 (70.3)	89 (67.9)	86 (65.6)	89 (65.9)	84 (66.7)	86 (65.2)

^a^Mycophenolate: mycophenolate mofetil or mycophenolate sodium

^b^Prednisone-equivalent per day. Denominator for this category is the number of patients with ≥ 1 oral corticosteroid in the time period

ACE, angiotensin-converting enzyme; ARB, angiotensin receptor blocker; IV, intravenous; NSAID, nonsteroidal anti-inflammatory drug

The mean (SD) number of visits to a rheumatologist among patients with utilization in this category was highest in Year 1 (3.1 [4.0]), then remained similar for Years 2–5 (Year 2: 2.3 [2.5]; Year 3: 2.1 [2.8]; Year 4: 1.8 [2.3]; Year 5: 1.8 [2.5]). A similar trend was observed for the number of follow-up visits to nephrologists (Year 1: 2.4 [4.7]; Year 2: 1.2 [3.4]; Year 3: 1.4 [4.1]; Year 4: 1.4 [4.1]; Year 5: 1.2 [3.3]), primary care physicians (Year 1: 12.7 [13.5]; Year 2: 9.1 [11.1]; Year 3: 8.9 [10.3]; Year 4: 10.0 [14.2]; Year 5: 10.4 [13.5]), and other provider specialties (Year 1: 48.8 [44.3]; Year 2: 38.1 [42.9]; Year 3: 38.9 [44.6]; Year 4: 43.4 [56.7]; Year 5: 43.1 [50.1]).

### SLE flares

Most patients (≥ 91.6%) had ≥ 1 SLE flare of any severity in each of the 5 years of follow-up (Table 4). The proportion of patients who experienced a severe SLE flare was higher in Year 1 (*n* = 106/335; 31.6%) than in subsequent years (14.3–18.5%), whereas the proportion of patients who experienced a mild SLE flare (50.8–58.2%) or a moderate SLE flare (85.1–90.2%) were more stable across each of the 5 years of follow-up.

**Table 4:** Counts and severity of SLE flares among patients with ≥ 5 years of follow-up (*n* = 335)

SLE flares^a^	Year 1	Year 2	Year 3	Year 4	Year 5
Patients with any SLE flare, n (%)	323 (96.4)	307 (91.6)	313 (93.4)	311 (92.8)	320 (95.5)
Total SLE flare count					
Mean (SD)	4.1 (2.1)	3.8 (2.2)	3.9 (2.2)	3.9 (2.3)	4.0 (2.2)
Median (IQR)	4.0 (3.0–6.0)	4.0 (2.0–5.0)	4.0 (2.0–5.0)	4.0 (2.0–5.0)	4.0 (2.0–6.0)
Patients with mild flare, n (%)	195 (58.2)	187 (55.8)	170 (50.7)	184 (54.9)	173 (51.6)
Mild flare count					
Mean (SD)	1.2 (1.3)	1.2 (1.4)	1.1 (1.4)	1.2 (1.4)	1.1 (1.3)
Median (IQR)	1.0 (0.0–2.0)	1.0 (0.0–2.0)	1.0 (0.0–2.0)	1.0 (0.0–2.0)	1.0 (0.0–2.0)
Patients with moderate flare, n (%)	302 (90.1)	285 (85.1)	290 (86.6)	285 (85.1)	295 (88.1)
Moderate flare count					
Mean (SD)	2.6 (1.7)	2.5 (1.7)	2.5 (1.7)	2.5 (1.8)	2.7 (1.7)
Median (IQR)	3.0 (1.0–4.0)	2.0 (1.0–4.0)	2.0 (1.0–4.0)	2.0 (1.0–4.0)	3.0 (1.0–4.0)
Patients with severe flare, n (%)	106 (31.6)	54 (16.1)	48 (14.3)	56 (16.7)	62 (18.5)
Severe flare count					
Mean (SD)	0.4 (0.7)	0.2 (0.5)	0.2 (0.6)	0.2 (0.6)	0.3 (0.7)
Median (IQR)	0.0 (0.0–1.0)	0.0 (0.0–0.0)	0.0 (0.0–0.0)	0.0 (0.0–0.0)	0.0 (0.0–0.0)

^a^Individual patients may have ≥ 1 flare

IQR, interquartile range; SD, standard deviation; SLE, systemic lupus erythematosus

### Healthcare costs

**Fig. 3:**
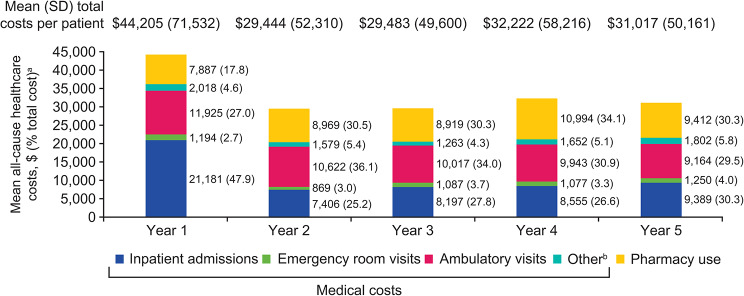
All-cause healthcare costs among patients with newly confirmed LN over 5 years of follow-up (*n* = 335) ^a^Numbers in parentheses are relative frequencies (percentages of the total costs in a given year for each cost component); ^b^Other medical costs include costs for services rendered at independent laboratories, at assisted living facilities, at urgent care clinics, and by home health providers. LN, lupus nephritis; SD, standard deviation

Total costs (medical and pharmacy, mean [SD]) per patient with a newly confirmed diagnosis of LN were higher in Year 1 ($44,205 [71,532]) than in subsequent years (Year 2: $29,444 [52,310]; Year 3: $29,483 [49,600]; Year 4: $32,222 [58,216]; Year 5: $31,017 [50,161]; Fig. 3). This difference was mainly driven by the cost of inpatient admissions, for which the mean (SD) costs were $21,181 (58,886) in Year 1 and lower in subsequent years (Year 2: $7,406 [23,331]; Year 3: $8,197 [24,110]; Year 4: $8,555 [27,403]; Year 5: $9,389 [29,283]; Additional file 1). In contrast, pharmacy costs were slightly lower in Year 1 (mean [SD]: $7,887 [18,337]) than in subsequent years (Year 2: $8,969 [26,886]; Year 3: $8,919 [30,245]; Year 4: $10,994 [37,919]; Year 5: $9,412 [31,079]).

## Discussion

This longitudinal cohort study conducted in the United States evaluated HCRU and healthcare costs of a sizeable, geographically diverse cohort of patients with a newly confirmed diagnosis of LN over a 5-year period. Patients with a newly confirmed diagnosis of LN were found to have high disease burden and were high utilizers of healthcare services, as evidenced by substantial HCRU, number of flares, and healthcare costs in each of the 5 years following their LN diagnosis. The burden was generally highest in the year following confirmed LN diagnosis and decreased, though remained high, in subsequent years.

Supporting the findings of the current study, previous studies have reported increased HCRU and healthcare costs in patients with SLE with LN compared with patients without LN or matched controls [21, 23–28]. Mean all-cause healthcare costs in the 12 months after diagnosis were significantly higher in patients with LN compared with patients with SLE alone ($50,975 vs. $26,262; *p* < 0.001) in a recent study by Bell et al. [28]. Clarke et al. compared annual direct medical costs of patients with varying degrees of renal damage and demonstrated that costs elevated with increasing degrees of renal damage [33]. Furthermore, Bell et al. demonstrated that patients with LN incur 2.2-fold greater healthcare costs per flare than patients without LN [34]. In a retrospective, claims-based analysis, Furst et al. demonstrated that a significantly higher proportion of patients with LN had utilization of ambulatory visits, ER visits, and inpatient admissions compared with patients without SLE [26]. Utilization of these healthcare resources and associated costs were all highest in the first year following LN diagnosis in the current study. Aghdassi et al. also demonstrated increased HCRU in patients with LN compared with patients with SLE alone [25]. In particular, patients with LN had more physician visits, diagnostic tests, hospital emergency visits, surgical procedures, and prescription and nonprescription medicines compared with patients with SLE without LN [25].

Furst et al. also reported that the increased annual healthcare costs observed in patients with LN compared with matched controls was predominantly driven by inpatient costs [26]. Similarly, Pelletier et al. reported that costs associated with inpatient and outpatient care were 252% and 146% higher, respectively, in patients with LN compared with patients with SLE without LN, with the increased total costs for patients with LN largely driven by inpatient hospitalization and outpatient services [23]. These findings are consistent with the current data where the high total costs in Year 1 were mostly driven by the cost of inpatient admissions. In addition, as Year 1 outcomes included the index claim where the LN diagnosis was captured, this may have contributed to the disproportionately high HCRU and costs observed in Year 1 versus the following years of follow-up.

As well as supporting previous findings of the high burden associated with LN, the current study also provides a longitudinal description of the HCRU and healthcare costs of LN over a period of 5 years following initial diagnosis. HCRU and costs were generally highest in the first year of follow-up. These findings contrast a previous 5-year analysis of a large Medicaid population with SLE and LN, which demonstrated higher HCRU and costs for patients with LN during their first year of follow-up compared with the following year; however, costs then increased to Year 5 [21]. A possible explanation for these findings is that patients with a newly confirmed diagnosis of LN initially require intensive medical care (e.g., the cost of diagnosis and induction therapy) and these costs generally decrease once the disease is stabilized, but may subsequently increase due to renal or extra-renal disease flares or infections [21].

The higher Year 1 costs found in this study may be due to a higher proportion of patients experiencing severe SLE flares in Year 1 compared with subsequent years. SLE flares have previously been associated with higher HCRU and costs when compared with patients without flares [35], and costs have been found to increase with flare severity [22]. Future analyses should include patients with SLE in the years prior to LN diagnosis to evaluate the trajectory of HCRU, costs, and number of flares as they progress to LN.

Strengths of this study include the use of commercial and Medicare Advantage insurance coverage data from newly diagnosed patients in the United States, compared with previous studies using data from either commercial or Medicaid populations [16, 21, 23, 24, 29], and its longitudinal nature, with the only other 5-year study utilizing older data from 1999 to 2005 [21].

Limitations of this study included the use of diagnostic codes in administrative claims data that do not guarantee that the patient had the disease, took certain medications, or underwent certain procedures. To mitigate the effects of this on the results, we used a previously validated claims definition to identify patients with LN [30], though the use of specific LN ICD-10 codes (M32.14 and M32.15) in the absence of other renal-related ICD-10 codes (e.g., end-stage kidney disease after SLE diagnosis) has not been fully evaluated. Finally, while the 12-month baseline period is greater than other studies in the literature [21, 23, 29, 36], it could not guarantee that patients were newly diagnosed with LN, as it is possible that patients had renal involvement but not a formal ICD diagnosis of LN. Herein lies one of the challenges of administrative claims analyses, in the reliance on ICD codes to identify diagnosed patients.

Results may not be generalizable to uninsured or Medicaid populations, as this study included a cohort of patients with commercial and Medicare Advantage insurance coverage only. In addition, the study excluded patients < 18 years of age; therefore, the HCRU and costs demonstrated here are not representative of those of the younger population of patients diagnosed with LN. The study also had a high survival bias (patient survival of 5 years post-index with continued healthcare enrollment was required), which could have led to underestimation of HCRU and costs. If variable lengths of follow-up had been allowed, higher accrued healthcare costs may have been reported due to changes in healthcare plan and coverage, and death. Finally, the lack of indirect cost data such as cost of loss of productivity, relapse, absenteeism, and short-term disability may limit understanding of the full scale of costs associated with LN.

## Conclusions

This study demonstrates substantial HCRU and healthcare costs of patients with LN over a period of 5 years following their confirmed diagnosis, with the highest burden experienced in the first year. This highlights the need for improved disease management to prevent renal damage, improve patient outcomes, and reduce costs among patients with renal involvement.

### Electronic supplementary material

Below is the link to the electronic supplementary material.

Supplementary Material 1

Supplementary Material 2

## Data Availability

The data that support the findings of this study are available from Optum but restrictions apply to the availability of these data, which were used under license for the current study, and so are not publicly available. Data are however available from the authors upon reasonable request and with permission of Optum. To request access to documents for this study, please submit an enquiry to Christopher F. Bell: christopher.f.bell@gsk.com.

## Abbreviations

ACE: Angiotensin-converting enzyme
ARB: Angiotensin receptor blocker
CKD: Chronic kidney disease
ER: Emergency room
HCRU: Healthcare resource utilization
ICD: International Classification of Diseases
IV: Intravenous
LN: Lupus nephritis
NSAID: Nonsteroidal anti-inflammatory drug
SD: Standard deviation
SLE: Systemic lupus erythematosus
