# Supplementary material for: Healthcare resource utilization and costs in patients with a newly confirmed diagnosis of lupus nephritis in the United States over a 5-year follow-up period
Source: BMC Health Serv Res. 2024 May 31;24:691. doi: 10.1186/s12913-024-11060-6 (PMC11143616; doi:10.1186/s12913-024-11060-6)
Supplement: Supplementary file 1 — Supplementary Material 1 [file 12913_2024_11060_MOESM1_ESM.pdf]

Additional file

Additional file 1 (.doc). Healthcare costs ($) among patients with a newly confirmed diagnosis of LN and at least 5 years of follow-up (n=335)

	**Year 1**	**Year 2**	**Year 3**	**Year 4**	**Year 5**
Medical costs ($)					
Inpatient admissions					
Mean (SD)	21,181 (58,886)	7,406 (23,331)	8,197 (24,110)	8,555 (27,403)	9,389 (29,283)
Median (IQR)	1,994 (0–19,516)	0 (0–0)	0 (0–0)	0 (0–3,207)	0 (0–787)
ER visits					
Mean (SD)	1,194 (2,536)	869 (2,477)	1,087 (2,877)	1,077 (2,702)	1,250 (3,236)
Median (IQR)	205 (0–1,277)	0 (0–612)	0 (0–820)	54 (0–882)	0 (0–862)
Ambulatory visits					
Mean (SD)	11,925 (27,798)	10,622 (29,077)	10,017 (21,413)	9,943 (20,801)	9,164 (17,807)
Median (IQR)	5,555 (2,388–11,589)	4,614 (1,848–9,315)	3,912 (1,970–9,953)	4,141 (1,696–9,494)	3,859 (1,865–8,167)
Other[a]					
Mean (SD)	2,018 (7,978)	1,579 (7,101)	1,263 (2,979)	1,652 (5,391)	1,802 (6,423)

Median (IQR)	590 (195–1,985)	374 (82–1,298)	331 (95–1,042)	351 (118–1,114)	385 (113–1,110)
Pharmacy costs ($)					
Mean (SD)	7,887 (18,337)	8,969 (26,886)	8,919 (30,245)	10,994 (37,919)	9,412 (31,079)
Median (IQR)	2,734 (1,154–6,647)	3,076 (1,291–6,373)	2,912 (1,262–6,328)	2,951 (1,283–6,832)	2,776 (1,178–6,466)

[a]Other medical costs include costs for services rendered at independent laboratories, at assisted living facilities, at urgent care clinics, and by home health providers.

ER, emergency room; IQR, interquartile range; LN, lupus nephritis; SD, standard deviation.
