# Supplementary material for: Healthcare resource utilization and costs in patients with a newly confirmed diagnosis of lupus nephritis in the United States over a 5-year follow-up period
Source: BMC Health Serv Res. 2024 May 31;24:691. doi: 10.1186/s12913-024-11060-6 (PMC11143616; doi:10.1186/s12913-024-11060-6)
Supplement: Supplementary file 2 — Supplementary Material 2 [file 12913_2024_11060_MOESM2_ESM.pdf]

Version 1

Reviewer 1:

Submitted: 11 Nov 2022

Feedback for the author(s)

Since decrease trends are described, tests for trend should be included, in order to properly interpret the results.

Is the index date included in 1st year?

If so, higher hospitalizations and ER visits costs could reflect the fact that index hospitalizations/ER are included in first year; I suggest, in this case, to describe index costs separated from 1st year.

Lin's method for censored costs (costs for patients with incomplete follow-up) would be more appropriate in presence of a so high rate of censored patients (84%); authors should do nothing, just my suggestion for eventual future papers.

Reviewer 2:

Feedback for the author(s)

1. Abstract

-The conclusion should reflect the primary objective and the results so not clear the relevance of this statement in the conclusion: “This highlights the need for interventions to prevent renal damage and improve outcomes in patients with renal involvement”

2. Methodology and results:

- This was a descriptive study and has no control arm (eg extra-renal or non-LN) so very limited conclusion can be drawn as no direct comparisons with other population could be made.

- There were lack of data on the detail medication cost that are commonly used in LN eg cyclophosphamide, mycophenolate, steroids, biologics (eg belimumab/rituximab) so very limited clinical implications can be derived from the results

3. Discussion

-Since this study was a descriptive study, the main discussion was based on other previous studies, in which majority had similar findings. Suggest to highlight any new knowledge from this study that may have an impact on the clinical practice or health policy

Editor

Submitted: 02 Jan 2023

Recommendation: Request Revision

The reviewers ask for adaption of several aspects. A broader consideration of the descriptive nature of the study appears to be necessary.

Version 2

Reviewer 2:

Submitted: 29 Mar 2023

Feedback for the author(s)

Thank you for the revision. The authors have addressed all the comments appropriately.

Reviewer 3:

Submitted: 01 May 2023

Feedback for the author(s)

Major:

1. The last line of the abstract Conclusion and the last line of the main text of the Conclusion does not directly relate to the interpretation of the study findings. I see this was revised but still does not directly relate to the study findings. You did not assess whether earlier diagnosis of LN would reduce HCRU compared with delayed diagnosis. Would revise this to directly relate to study findings.

2. The one-year observational period prior to the first LN related code may not be long enough to ensure that these are all new-onset LN. It is possible some of these patients had prevalent LN but had codes fewer than once per year. To identify patients with an incident diagnosis, other studies have used a minimum of 2 years of enrollment/observation prior to the first LN-related code (Ref: Yazdany J et al. Quality of care for incident lupus nephritis among Medicaid beneficiaries in the United States. Arthritis Care Res (Hoboken). 2014 Apr;66(4):617-24)), and some studies have required 6 years of enrollment/observation prior to the first code (i.e., the Population BC SLE inception cohort). This is a major concern given that this study focuses on “newly confirmed LN.” Would consider revising the analysis to require 2 years of observation prior to the index date of the first LN-related code. The low utilization of mycophenolate and cyclophosphamide in Table 3 also makes me suspect some of these patients are prevalent cases of LN and/or some patients are misclassified by non-specific nephritis codes.

3. Has the definition of LN used in this study been previously validated? There are validation studies of ICD-9 based LN codes (Chibnik et al, 2010). However, I am not aware of a study validating the use of ICD-10 based codes for the diagnosis of LN and there is some variation in which non-specific nephritis-related ICD-10 codes are used. Would cite such a validation study if present or state as a limitation that the LN diagnosis algorithm has not been validated.

4. This sentence about provider specialty making the LN diagnosis is unclear: “Overall, 38% of patients (n=820/2,159) received their index diagnosis of LN from physician specialties other than primary care physicians/family practitioners/internal medicine (716/2,159; 33.2%), nephrologists (390/2,159; 18.1%), or rheumatologists (233/2,159; 10.8%).” Does this mean 10.8% of the diagnoses came from rheumatologists or that 10.8%? Sentence structure would imply 10.8% were not diagnosed by a rheumatologist. If I interpret the correct intent, were 67% diagnosed by primary care and 33% diagnosed by other which included 18.1% by nephrology and 10.8% by rheumatology? Would rephrase.

5. For Figure 2, the Y axis label seems incorrect/unclear. Is this supposed to be the percentage of patients who were followed for 5 years who had each of the 4 types of health care costs in each year? Would clarify the label.

6. Can you provide details about cost according to the LN treatment regimen? (I.e., induction therapy with mycophenolate vs IV cyclophosphamide? What about belimumab use?

7. I agree with the prior reviewer that it would strengthen this study to apply statistical tests when comparing the rates of events between different numbers of years from the index date.

8. It would be helpful to state in the text that the index encounter when the LN diagnosis was captured was included in the year 1 costs. Could mention in the discussion that the higher Year 1 costs could be driven by index hospitalizations when the diagnosis of LN was made.

9. Did you capture costs from renal replacement therapy including dialysis and kidney transplants? It would be interesting if you could indicate what role that adverse outcome from LN had on HCRU since you have 5 years of follow up data.

10. Rather than stating as a limitation in the discussion that your Table 1 patient characteristics are for all patients with 1 year of follow up rather than our primary analytic population with 5 years of follow up, why not include a column in that table with the baseline characteristics of patients with 5 years of follow up?

Minor:

1. The first line of the methods is an incomplete sentence. Since this section is titled “Background” rather than “Objective”, would be clearer to rephrase to something like “We aimed to describe…”

2. In the Abstract Results section, there seems to be a typo in the sentence “Among patients with HCRU…” Did you mean “among patients with LN”?

3. In the Background, would clarify in the text the setting for reference 17, since HCRU in SLE patients varies between Medicare, Medicaid, and commercially insured populations.

Editor:

Submitted: 14 Jun 2023

Recommendation: Request Revision

Please find the very detailed reviewers feedback at the end of this email and note that as an original reviewer was unavailable for re-review, we sought the advice of an additional reviewer on your revisions. We kindly ask that you address these comments carefully and respond to all reviewers' comments in your point-by-point response, indicating where changes to the manuscript have been made (e.g. page, line). We recommend highlighting all the changes you apply to the manuscript or enabling the track changes functionality.

Please also accept our apologies for the delays you have experienced with your manuscript.

Version 3

Reviewer 4:

Submitted: 02 Oct 2023

Feedback for the author(s)

"PEER REVIEWER ASSESSMENTS:

OBJECTIVE - Full research articles: is there a clear objective that addresses one or several testable research questions? (Brief or other article types: is there a clear objective?)

Yes - there is a clear objective

DESIGN - Is the current approach (including controls and analysis protocols) appropriate for the objective?

Yes - the approach is appropriate

EXECUTION - Are the experiments and analyses performed with sufficient technical rigor to allow confidence in the results?

Yes - experiments and analyses were performed appropriately

STATISTICS - Is the use of statistics in the manuscript appropriate?

Yes - appropriate statistical analyses have been used in the study

INTERPRETATION - Is the current interpretation/discussion of the results reasonable and not overstated?

Yes - the author's interpretation is reasonable

OVERALL MANUSCRIPT POTENTIAL - Has the author addressed your concerns sufficiently for you to now recommend the work as a technically sound contribution? If not, can further revisions be made to make the work technically sound?

Yes - current version is technically sound

PEER REVIEWER COMMENTS:

GENERAL COMMENTS: Overall, I think the authors had addressed the critiques from previous reviewer 3 in a professional manner. They had either incorporated comments received from the reviewer in the revised manuscript or made adequate clarifications when they did not do so. I enjoy reading the rebuttal letter, indeed. With all that said, I have some suggestions for the authors to consider as noted below.

1. Judging from Tables 2 and 4 as well as additional file 1 it is palpable that numerous outcome measures were skewed to the right since presented standard deviation (SD) values were quite large, even larger than associated mean values. Such results are expected, indeed. Still, it would be more preferable that the authors supply median and range values of those outcome measures, in addition to mean and SD statistics.

2. Figure 1: The current figure title is “Study design”. However, I do not think the contents of that figure are related to any kind of study design (e.g., the randomized controlled trial); rather, it illustrates the data collection period per se.

3. Table 1: “(n=335)” appeared twice, redundantly.

4. Lastly, all tables need to be modified to be shown in a more professional fashion. For instance, the table contents are narrower than the width of the table title for both Tables 1 and 4, and the horizontal line is missing at the bottom of Table 1.

Editor:

Sent: 16 Oct 2023

Decision: Revision Requested

Your manuscript, "Healthcare Resource Utilization and Costs in Patients With a Newly Confirmed Diagnosis of Lupus Nephritis in the United States Over a 5-year Follow-up Period", has now been assessed.

Please note that as one of the original Reviewers was unavailable for re-review, we sought the advice of an additional Reviewer on your revisions.

This Reviewer has now raised a few outstanding comments that would need to be addressed before we can proceed with your submission. You will find the full report below.

Thus, we invite you to revise your paper. Please carefully respond to all remaining Reviewers' comments in your response letter and in a point-by-point manner, indicating where changes to the manuscript have been made (e.g. page, line). We also recommend highlighting all the changes you apply to the manuscript or enabling the track changes functionality.

When your revision is ready, please submit the updated manuscript and a point-to-point response if applicable.

Version 4

Reviewer 4:

Submitted: 13 Dec 2023

Feedback for the author(s)

PEER REVIEWER ASSESSMENTS:

OBJECTIVE - Full research articles: is there a clear objective that addresses one or several testable research questions? (Brief or other article types: is there a clear objective?)

Yes - there is a clear objective

DESIGN - Is the current approach (including controls and analysis protocols) appropriate for the objective?

Yes - the approach is appropriate

EXECUTION - Are the experiments and analyses performed with sufficient technical rigor to allow confidence in the results?

Yes - experiments and analyses were performed appropriately

STATISTICS - Is the use of statistics in the manuscript appropriate?

Yes - appropriate statistical analyses have been used in the study

INTERPRETATION - Is the current interpretation/discussion of the results reasonable and not overstated?

Yes - the author's interpretation is reasonable

OVERALL MANUSCRIPT POTENTIAL - Has the author addressed your concerns sufficiently for you to now recommend the work as a technically sound contribution? If not, can further revisions be made to make the work technically sound?

Yes - current version is technically sound

PEER REVIEWER COMMENTS:

GENERAL COMMENTS: I appreciate that the authors paid heed to all my suggestions, and revised their manuscript accordingly. The revised manuscript has been clearer and strengthened by the revisions. I think this work is ready to be published.

Editor:

Sent: 18 Dec 2023

Decision: Revision Requested

Major

Hide revision request

Comments to the authors

Your manuscript, "Healthcare Resource Utilization and Costs in Patients With a Newly Confirmed Diagnosis of Lupus Nephritis in the United States Over a 5-year Follow-up Period", has now been assessed. Based on the comments below, we note that the manuscript has significantly improved to the publishable level. However, we would like to ask for further amendments regarding the rationale of this study, particularly in comparison with previously published results (e.g. Bell CF, Wu B, Huang SP, Rubin B, Averell CM, Chastek B, Hulbert EM, Von Feldt J. Healthcare Resource Utilization and Associated Costs in Patients With Systemic Lupus Erythematosus Diagnosed With Lupus Nephritis. Cureus. 2023 Apr 19;15(4):e37839. doi: 10.7759/cureus.37839. PMID: 37214060; PMCID: PMC10198302.). We feel that the net advance represented by the current manuscript under consideration at the journal would need to be highlighted in the main text. Thus, we recommend revising the 'Background' and 'Discussion' sections of the manuscript to clarify how this study contributes to the literature and how it builds on work that was previously published. We would like to remind you also that, according to our policies here: https://www.biomedcentral.com/getpublished/editorial-policies#duplicate+publication, it is mandatory to adequately cite prior works in all the appropriate sections. We feel this is necessary to adequately define the net contribution that this work makes to the literature and to strengthen the rationale of this study. Please respond to all comments in your point-by-point letter and indicate where changes to the manuscript have been made (e.g. page, line). We also recommend highlighting all the changes you apply to the manuscript or enabling the track changes functionality to facilitate reassessment.
